# E-vape and E-Cigarettes-Associated Lung Injury (EVALI) in the COVID-19 Pandemic: A Diagnostic Dilemma and Therapeutic Challenge

**DOI:** 10.7759/cureus.26200

**Published:** 2022-06-22

**Authors:** Gaurav Mandal, Ajit Lale, Rick Greco

**Affiliations:** 1 Internal Medicine, Trinity Medical Center West, Steubenville, USA

**Keywords:** systemic steroids, social history, acute hypoxemic respiratory failure, covid-19, evali

## Abstract

E-vape and e-cigarettes-associated lung injury (EVALI) is a diagnostic dilemma and even more obscure during the coronavirus disease 2019 (COVID-19) pandemic. A rise was seen in EVALI cases at the beginning of the COVID-19 pandemic. Still, the non-specific presentation, or the overlapping symptoms of COVID-19 and EVALI, can negate the possible diagnosis of EVALI because of a clinician's predisposition toward infectious etiologies, and it becomes even more challenging during a viral pandemic. The patient's social history remains the key distinctive point in diagnosing EVALI. Systemic steroids are generally used along with supportive care to treat patients with EVALI. This case report demonstrates the dilemma in diagnosing EVALI in a 19-year-old female during the COVID-19 pandemic.

## Introduction

There has been heavy marketing of cigarette alternatives since their launch in the United States of America (USA) in 2007 [1]. E-cigarettes are devices that aerosolize nicotine-based or cannabis-based concentrates mixed with other solvents. E-vape and e-cigarettes-associated lung injury (EVALI) is an inflammatory response in the lungs from the inhalation of vapes formed from the products in e-cigarettes and should be suspected with use within 90 days of symptom onset [2]. EVALI is particularly prevalent among the youth [2]. Patients with EVALI may experience life-threatening hypoxemia, requiring critical monitoring [1]. In late 2019 and early 2020, there have been EVALI outbreaks and increased hospitalizations from EVALI in the USA [2-3]. Also, during this period, the coronavirus disease 2019 (COVID-19), caused by severe acute respiratory syndrome coronavirus 2 (SARS-CoV-2), became a significant public health concern that later evolved into a pandemic [4]. During the COVID-19 pandemic, multiple case reports have demonstrated overlapping symptoms in patients with COVID-19 and EVALI [5]. Without prior exposure to this knowledge, we also encountered a similar case. In this case report, we came across a 19-year-old female who presented to the emergency department (ED) during the first wave of the COVID-19 pandemic with complaints of shortness of breath, fever, chills, cough, and diarrhea.

## Case presentation

**Figure 1:**
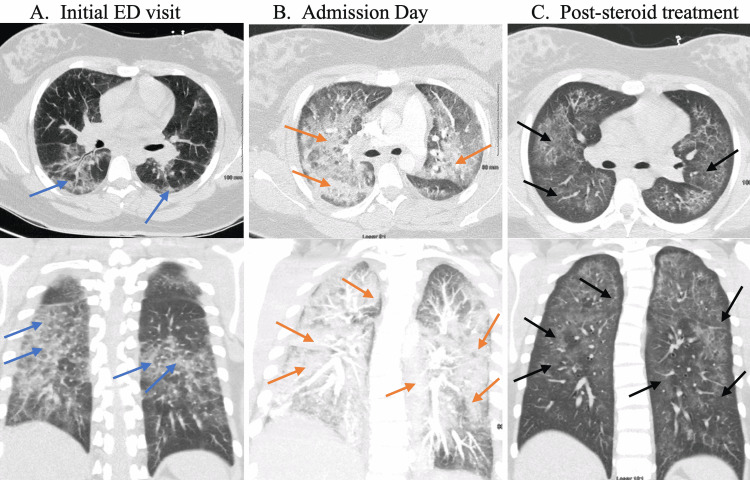
Computed tomography findings A. Initial ED visit: Ground-glass infiltrates in multiple areas in the bilateral upper and lower lobes, along with some regions of peri-bronchial thickening and some infiltrate radiating from the hila (blue arrows). B. Admission day: Worsening bilateral coalescent alveolar infiltrates (red arrows) compared to the initial visit. C. Post-steroid treatment: Marked decrease in the infiltrates (black arrows) 48-hour post-high-dose intravenous steroid treatment. ED: emergency department

A 19-year-old female presented to the ED with recurrent shortness of breath. She endorsed shortness of breath at rest, pleuritic chest pain, productive cough, chest congestion, fever, myalgia, nausea, anorexia, non-bloody emesis, and non-bloody diarrhea. Three days before this presentation, she had tested negative for the SARS-CoV-2 polymerase chain reaction (PCR) test and was sent home from the ED with a five-day course of oral azithromycin and a presumptive diagnosis of atypical pneumonia. At that time, a chest computed tomography (CT) scan showed ground-glass infiltrates in multiple areas in the bilateral upper and lower lobes, along with some regions of peri-bronchial thickening and some infiltrate radiating from the hila (Figure 1). However, her respiratory distress worsened at home, which rendered her back to the hospital. She had a past medical history of attention deficit hyperactivity disorder (ADHD). She denied any combustible smoking.

On physical examination, she was an obese Caucasian female in moderate distress, with a temperature of 101.6 degrees Fahrenheit, pulse rate of 126 beats per minute, respiratory rate of 38-48 breaths per min, blood pressure of 117/60 mm Hg, and O2 saturation of 90% on 5 liters per minute. There were diffuse mild expiratory wheezes on lung auscultation. The remainder of the examination was ordinary.

Her labs were remarkable for a white blood cell (WBC) count of 15,800 with neutrophilia of 89.5%, lymphopenia of 5.2%, and an absolute lymphopenia of 800 per milliliter. Potassium was 3.1, and arterial blood gas showed respiratory alkalosis and hypoxemia with a partial pressure of oxygen of 60.8 mmHg on 5 liters of oxygen per minute. A repeat SARS-CoV-2 PCR test was negative. Her urine toxicology screen was positive for cannabinoids (Table 1). The D-dimer level was 2.57 mg/L fibrinogen equivalent unit, and her CT angiography scan of the chest showed a significant increase in the bilateral coalescent alveolar infiltrates compared to the findings three days ago and was negative for pulmonary embolism (Figure 1). She was admitted for acute hypoxic respiratory failure and suspected severe sepsis from possible community-acquired pneumonia and was started empirically on intravenous (IV) ceftriaxone and IV azithromycin.

**Table 1:** Patient’s laboratory data on hospital Day 1 PE/DVT: pulmonary embolism/deep vein thrombosis; FEU: fibrinogen equivalent unit; pCO2: partial pressure of carbon dioxide; pO2: partial pressure of oxygen

Variable	Reference Range, Adults	Patient’s labs
White cell count (per ml)	4500-11,000	15,800
Neutrophils%	40.0-80.0%	89.5
Lymphocyte%	13.0-47.0%	5.2
Lymphocyte (per ml) #	1200-5200	800
Sodium (mmol/liter)	136-145	136
Potassium (mmol/liter)	3.5-5.1	3.1
Carbon dioxide (mmol/liter)	21-32	27
Creatinine (mg/dl)	0.55-1.02	0.72
Magnesium (mg/dl)	1.6-2.6	1.7
Albumin (g/dl)	3.5-5.0	2.7
Lactic acid (mmol/liter)	0.4-2.0	1.4
Urine cannabinoids (ng/ml)	Not Detect	Present, 197
D-dimer Quant PE/DVT (mg/liter FEU)	<0.5	2.57
Arterial pH	7.35-7.45	7.491
Arterial pCO2 mmHg	35-45	32.6
Arterial pO2 mmHg	80-100	60.8
Streptococcus pneumoniae antigen, urine	Not Detected	None Detected
Legionella pneumophila antigen, urine	Not Detected	None Detected

On Day 2 of the hospital stay, she continued to have a productive cough with occasional hemoptysis. She continued to endorse dyspnea at rest and chest congestion. She remained tachypneic and tachycardic. Her oxygen requirement increased to 12-15 liters per minute to maintain an oxygen saturation just above 90% at rest. Her oxygen saturation dropped to 70-84% with minimal ambulation out of bed. Due to her lack of symptom improvement and being in the era of the COVID-19 pandemic, we opted to do another SARS-CoV-2 PCR test, which was negative for the third time in one week (Table 2). It was impossible to wean the patient off the oxygen, and inhaled bronchodilators were ineffective in alleviating her respiratory distress. By Day 3, the patient's WBC count improved, and she became afebrile, but there wasn’t any improvement in her dyspnea on exertion, tachypnea, or oxygen requirement. The test results of urinary Streptococcus pneumoniae Ag, urinary Legionella pneumophila Ag, and blood cultures were negative.

**Table 2:** Patient’s RT-PCR tests to detect SARS-CoV-2 RNA from the upper respiratory tract RT-PCR: reverse-transcription polymerase chain reaction; SARS-CoV-2: severe acute respiratory syndrome coronavirus 2; RNA: ribonucleic acid

Nasal swab specimen collection date	Reference Range	Result
Three days before admission	Not Detected	Not Detected
Hospital Day 1	Not Detected	Not Detected
Hospital Day 3	Not Detected	Not Detected

We continued to monitor her progress, but we decided to revisit her diagnosis given the treatment failures. Upon further interviewing and exploring her social history, the patient stated that she had frequently used multiple vaping appliances daily over the past five years, with active ingredients being nicotine and tetrahydrocannabinol (THC). She had been trying out the newer products on the market and used them until her initial ED visit. EVALI was strongly suspected given her recent vaping, presenting symptoms, respiratory failure, and airspace disease on the CT scans [1]. Given her life-threatening hypoxia, we started her on high-dose IV steroids with methylprednisolone 1 mg/kg daily. With this regimen, we observed a gradual recovery of her respiratory status within the next 36 hours, and she was off any oxygen requirement. She did not qualify for home oxygen with the six-minute walk test and was sent home on a tapering dose of oral steroids. Before discharge, we counseled our patient on the ill effects of e-cigarettes and educated her on her diagnosis of EVALI.

## Discussion

There has been an increase in the use of vaping products in the 2010s decade. The use of e-cigarettes substantially increased from 2011 to 2018, from 7 million to 41 million users, with the most dramatic rise in middle and high school students [6]. The non-specific findings and symptoms render EVALI a diagnosis of exclusion and clinical probability [7]. However, EVALI should be strongly suspected when the patient has a history of E-vape and e-cigarettes use in the past and, more importantly, within the past month [1].

A patient with EVALI typically presents with severe respiratory distress [1]. Symptoms include, but are not limited to, cough, shortness of breath, pleuritic chest pain, nausea, vomiting, diarrhea, fatigue, fever and chills, and weight loss [8]. Laboratory findings are relatively non-specific, demonstrating elevation in WBC count with a neutrophilic predominance and an increase in the levels of inflammatory markers such as erythrocyte sedimentation rate, C-reactive protein, ferritin, D-dimer, and lactate dehydrogenase [9-10]. Chest X-ray typically shows bilateral infiltrates, and the predominant CT finding remains bilateral ground-glass opacification, observed in all patients [1]. Although there is no universal established diagnostic algorithm for EVALI, due to the apparent septic presentations of these patients, the typical diagnostics involve infectious workups that include blood cultures, respiratory viral panel, Streptococcus and Legionella antigens, mycoplasma antibody, and human immunodeficiency virus (HIV) tests, along with detailed exploration of relevant social history and environmental exposures [1].

The most common pathologic findings of EVALI include organizing pneumonia and diffuse alveolar damage, and the less common are eosinophilic pneumonia and diffuse alveolar hemorrhage [3]. Hypersensitivity pneumonitis is another pattern of lung injury seen in these patients [3,11]. Vitamin E and THC have been consistently observed in EVALI cases, and these are more linked to THC-containing e-liquids than nicotine-containing e-liquids [12]. In a report from the Center for Disease Control and Prevention (CDC), bronchoalveolar lavage (BAL) commonly detected vitamin E in all patient samples (n =29). Currently, the data favors more association than causation between vitamin E and EVALI [13]. BAL also shows lipid-laden macrophages in most patients. However, this finding is not sensitive or specific enough to exclude or include EVALI [14]. The pathophysiology is yet to be fully discovered, but there could be potential inhalational injuries from flavorants, volatile organic compounds, and heavy metals [11].

The clinical course of patients with EVALI varies. Almost half of the patients with EVALI may require high amounts of oxygen that meet the Berlin definition of acute respiratory distress syndrome [3]. About a half to two-thirds of the hospitalized patients with EVALI may require admission to the intensive care unit [1,3]. In addition, pneumothorax, pleural effusions, and pneumomediastinum complications have also been reported [3]. Early determination of this disease is crucial, as the respiratory status may worsen to the point of requiring invasive ventilation or extracorporeal membrane oxygenation, and fatalities have also occurred [14-15].

There is a diagnostic dilemma of EVALI during the COVID-19 pandemic. The similarities in presenting symptoms, progression of the disease, and imaging findings make it challenging to distinguish between EVALI and COVID-19 [10,15]. Also, patients tend to initially withhold valuable information about their exposure to e-cigarettes, as encountered in our case, likely from the fear of being judged, embarrassment, or hesitancy to hear about their harmful behavior [15]. The cases of EVALI have increased at a higher rate during the COVID-19 pandemic [7]. As such, it should not be excluded from our differential diagnoses. In addition, COVID-19 also confounds the diagnosis of EVALI and, in turn, leads clinicians to extensive unrevealing workups, which could delay the treatment of EVALI [7]. Still, ruling out SARS-CoV2 infection is crucial during this COVID-19 pandemic, and patients typically receive empiric antibiotics for possible infectious etiologies [7]. To improve our diagnostic yield, we could have investigated with a radiologist regarding the sparing of the subpleural spaces that are often appreciated in CT imagings in EVALI patients, which may have aided in diagnosing our patient sooner [1,5]. Also, we could have ruled out other respiratory viral etiologies. Despite these shortcomings, this case further raises the importance of a clinician inquiring about e-cigarette use in patients with acute lung disease.

Some differentiating features between EVALI and COVID-19 have been reported so far. In terms of laboratory data, patients with EVALI typically have neutrophilic leukocytosis while COVID-19 patients have normal to low WBC count and lymphopenia [7,10]. However, our case had a mixed picture of both diseases in terms of these parameters, which had also been documented previously [10]. Epidemiologically, only in rare circumstances are youths severely affected with COVID-19, unlike the general trend observed with EVALI [7]. Also, EVALI and COVID-19 respond differently to treatment with systemic steroids. Patients with EVALI respond much quicker, typically within one to three days, with a drastic improvement in respiratory symptoms and oxygen requirement. Those with COVID-19 pneumonia respiratory failure, however, historically required a longer steroid course duration and had prolonged recovery times following critical illness [7,16]. For immunomodulation in COVID-19 patients, the effectiveness of dexamethasone 6 mg daily has been well-validated [17]. But, the evidence-based treatment guideline to manage lung injury and systemic inflammation in EVALI patients is yet to be established. So far, multiple case series of EVALI have demonstrated clinical improvement with systemic steroid doses such as prednisone 40 mg orally daily or methylprednisolone 120 mg to 500 mg IV daily [3,9,14,18]. Some experts also recommend a short course of a moderate dose of steroids with prednisone 40-60 mg orally daily for five to 10 days [7].

## Conclusions

Clinicians need to recognize the importance of taking a relevant social history, including inquiries about e-cigarette and vaping product use. Even though there is a rise in the use of e-cigarettes, the diagnosis of EVALI can be missed or delayed during the COVID-19 pandemic. Even though there are some distinctions between the two diseases, due to the many overlapping features between EVALI and COVID-19, the diagnostic dilemma is primarily driven by patients not volunteering their vaping history and thus withholding this valuable information from the clinicians. In addition, a clinician's predisposition to the common diagnoses catalyzed by a viral pandemic further increases the diagnostic complexity. A high index of suspicion of EVALI must be maintained among clinicians after ruling out COVID-19, particularly when patients do not respond to antibiotics. Finally, this case highlights the diagnostic difficulties in distinguishing EVALI from infectious lung pathologies and how it can delay the treatment.
